# Analysis of the mandibular canal course using unsupervised machine learning algorithm

**DOI:** 10.1371/journal.pone.0260194

**Published:** 2021-11-19

**Authors:** Young Hyun Kim, Kug Jin Jeon, Chena Lee, Yoon Joo Choi, Hoi-In Jung, Sang-Sun Han

**Affiliations:** 1 Department of Oral and Maxillofacial Radiology, Yonsei University College of Dentistry, Seoul, Republic of Korea; 2 Department of Preventive Dentistry & Public Oral Health, Brain Korea 21 PLUS Project, Yonsei University College of Dentistry, Seoul, Republic of Korea; 3 Clinical Imaging Data Science (CCIDS), Yonsei University College of Medicine, Seoul, Republic of Korea; Sun Yat-Sen University, CHINA

## Abstract

**Objectives:**

Anatomical structure classification is necessary task in medical field, but the inevitable variability of interpretation among experts makes reliable classification difficult. This study aims to introduce cluster analysis, unsupervised machine learning method, for classification of three-dimensional (3D) mandibular canal (MC) courses, and to visualize standard MC courses derived from cluster analysis in the Korean population.

**Materials and methods:**

A total of 429 cone-beam computed tomography images were used. Four sites in the mandible were selected for the measurement of the MC course and four parameters, two vertical and two horizontal parameters were measured per site. Cluster analysis was carried out as follows: parameter measurement, parameter normalization, cluster tendency evaluation, optimal number of clusters determination, and k-means cluster analysis. The 3D MC courses were classified into three types with statistically significant mean differences by cluster analysis.

**Results:**

Cluster 1 showed a smooth line running towards the lingual side in the axial view and a steep slope in the sagittal view. Cluster 2 ran in an almost straight line closest to the lingual and inferior border of mandible. Cluster 3 showed the pathway with a bent buccally in the axial view and an increasing slope in the sagittal view in the posterior area. Cluster 2 showed the highest distribution (42.1%), and males were more widely distributed (57.1%) than the females (42.9%). Cluster 3 comprised similar ratio of male and female cases and accounted for 31.9% of the total distribution. Cluster 1 had the least distribution (26.0%) Distributions of the right and left sides did not show a statistically significant difference.

**Conclusion:**

The MC courses were automatically classified as three types through cluster analysis. Cluster analysis enables the unbiased classification of the anatomical structures by reducing observer variability and can present representative standard information for each classified group.

## Introduction

Assessing the course of the mandibular canal (MC) is one of the major considerations for dentists in that the MC contains important anatomical structure, inferior alveolar neurovascular bundle, that provides sensation and blood to the mandible [1, 2]. Although clinicians carefully treat patients to avoid nerve injury, the inferior alveolar nerve is still commonly damaged during various dental procedures, such as third molar extraction, implant surgery, local anesthesia injection, or endodontic treatment [3–5]. Temporary or permanent complications of the nerve injury can significantly reduce the patient’s physical and mental quality of life [6, 7]. Accordingly, a better understanding of the MC would be a key factor in successful dental treatment.

Many researchers have used dry skulls and radiographs for the MC course classification despite some notable limitations [8–13]. For example, a limited number of dry skulls may not sufficiently reflect the anatomical variations of the MC courses, and the absence of relevant information such as sex, age, and disease history make it difficult to obtain accurate results [13, 14]. With the development of dental imaging modalities such as panoramic radiograph and CBCT, researchers have begun evaluating more cases than those used in cadaver dissection studies to classify MC course [9, 13]. However, the number of the MC courses reported in previous studies has inconsistency with two to four types. This discrepancy may be due to the inevitable variability among researchers when interpreting and classifying anatomical structures [15]. It is another drawback that the most studies have classified the MC courses from a three-dimensional (3D) perspective. In other words, previous studies have been analyzed the location of the MC using two-dimensional (2D) panoramic image, or even if they used CBCT, the MC course was derived in one view such as horizontal or vertical. Therefore, to overcome these shortcomings, it is necessary to introduce a new approach that enables objective classification of the 3D anatomical structure without observer bias.

Cluster analysis, one of unsupervised machine learning techniques for data mining, is a useful method to categorize large data into clusters based on the similarities or distances (dissimilarity) among individual objects [16]. In the dental field, this statistical approach has been used to determine the size of the replacement prosthesis for temporomandibular joint, analyze dental caries data, and identify inequality in oral healthcare approaches in adults [14, 17, 18]. The clustering process continues until the distance between objects assigned to one cluster is minimized and the distance between each separated cluster is maximized. Eventually, clusters classified as a result of this machine learning technique will ultimately have different characteristics. Based on this concept, when information on the anatomical structure of medical images is analyzed, it is possible to obtain objective results with minimal subjective judgment. To the best of our knowledge, however, no studies have been applied cluster analysis to classify the normal anatomy of the mandibular canal.

Hence, the goal of this study is to introduce cluster analysis to classify 3D MC courses in the Korean population, and to visualize standard MC courses from the classified results.

## Materials and methods

### Data preparation

Cone-beam computed tomography (CBCT) images of 429 Korean patients were selected from the picture archiving and communication system of Yonsei University Dental Hospital. Patients with pathologic lesions, orthognatic surgery history, or complete edentulous, and unclear images with blurring or artifacts were excluded from the data. All CBCT images were acquired by RAYSCAN Alpha Plus device (Ray Co. Ltd, Hwaseong-si, Korea) using the following acquisition settings; field of view, 16 x 10 cm; voxel size, 0.23 mm; 90 kVp; 12 mA; and exposure time, 14s. Selected data were anonymized and exported in digital imaging and communication in medicine (DICOM) format for analysis.

This study was approved by the Institutional Review Board (IRB) of Yonsei Dental Hospital (No.2-2019-0073) and were performed in accordance with relevant guidelines and ethical regulations. The IRB of Yonsei Dental Hospital granted a waiver of informed consent form due to the retrospective nature of this study.

### Mandibular canal course classification

**Fig 1:**
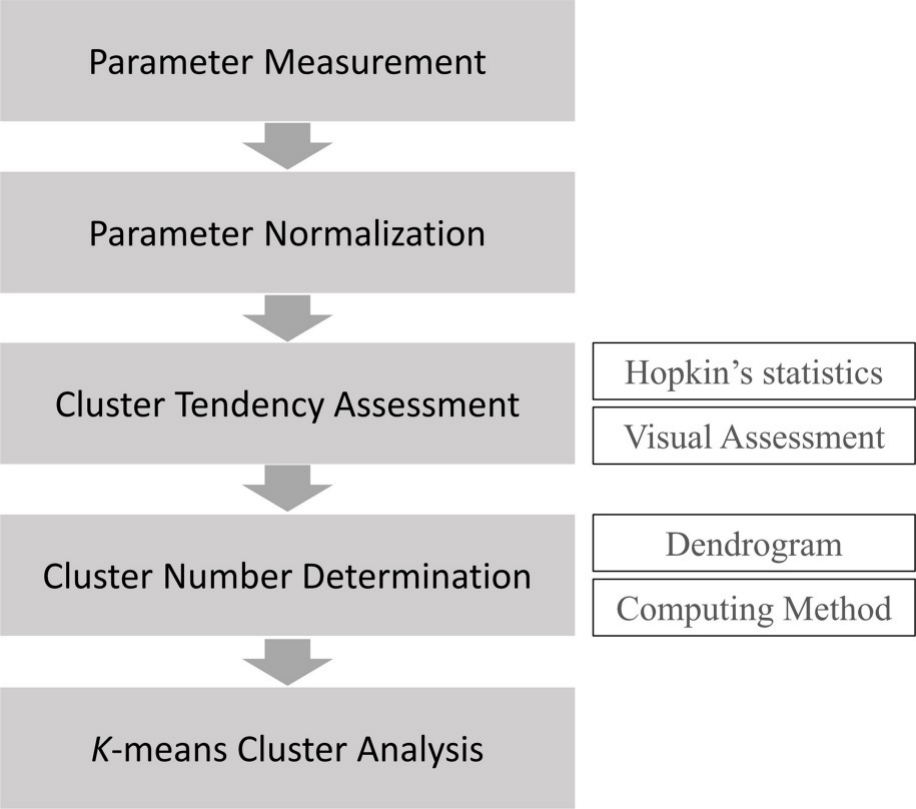
The overall workflow of cluster analysis.

Fig 1 shows the overall workflow of cluster analysis.

**Fig 2:**
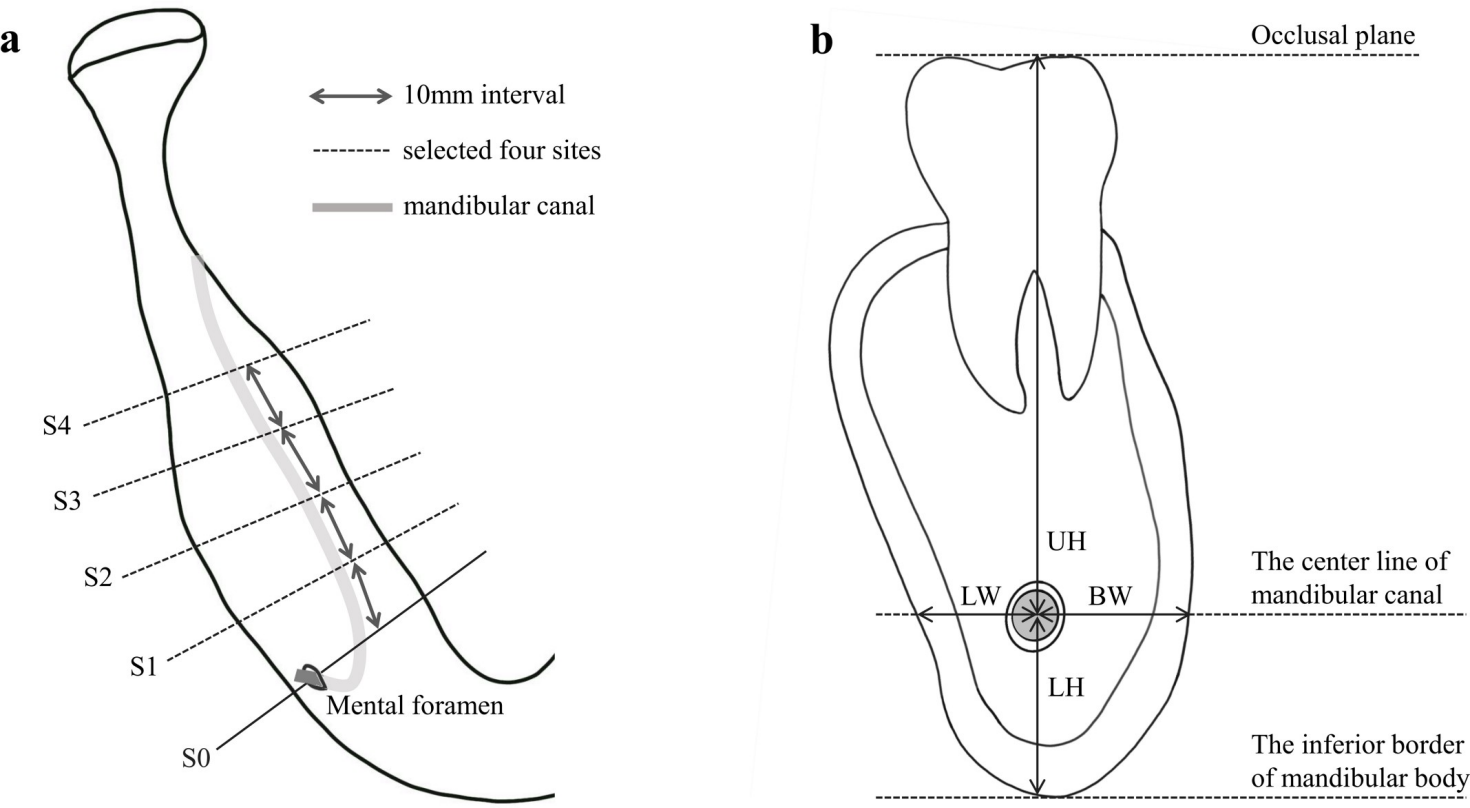
Two schematic diagrams illustrating the four selected sites and parameters. (a) Selection of four sites in a CBCT image. S0 is the baseline that penetrates the center of mental foramen. Four cross-sectional images (S1, S2, S3 and S4) were selected at intervals of 10mm from S0. (b) Four parameters—upper height (UH), lower height (LH), lingual width (LW), and buccal width (BW)–used to assess the course of the mandibular canal. The mandibular canal is represented in gray.

In the first stage, CBCT images were re-oriented using the mandibular occlusal plane, and were reconstructed automatically at the axial level where root furcation of the first molar existed. Four sites (S1, S2, S3 and S4) were selected at 10mm intervals from the center of the mental foramen (S0) (Fig 2A). In each site, two vertical parameters, upper height (UH) and lower height (LH), and two horizontal parameters, buccal width (BW) and lingual width (LW), were measured in millimeter (Fig 2B). All measurement workflow was conducted using Ondemand3D software (Cybermed Inc., Seoul, Korea). To ensure the reliability of the measured parameters, 30 CBCT images were randomly selected and measured by two observers. The intra-class correlation coefficients (ICCs) were conducted with 95% confidence interval (CI) and was reported as 0.983 for all measurements.

In the following step, since the location of the MC course can be specified in the form of coordinates by two parameters, one vertical and one horizontal parameter, LH and BW, were selected and converted into ratio values for normalization. Increase in the LH and BW ratio imply that the MC position is closer to the occlusal plane and lingual border, respectively. Subsequently, cluster tendency of the parameters was assessed with Hopkin’s statistics [19] and Visual Assessment of cluster Tendency (VAT) algorithm [20] to determine the propriety of performing cluster analysis on the normalized parameters. The Euclidean distance, which is the most widely used method for ratio data, was applied to calculate the dissimilarity between pairs of parameters [21]. Once clusters are found in the data set, it is necessary to determine the optimal number of clusters. Dendrogram, the output of a hierarchical clustering algorithm, is a tree diagram showing the relationship between parameters. In this study, dendrogram was generated using the Ward linkage method, which minimizes the total within-cluster variance [22], and the observer subjectively estimated the optimal number of clusters primarily. To objectively verify the number of clusters, computing method, NbClust, was then applied. NbClust is a useful package that proposes the best number of clusters by computing all combinations of clustering methods and distance measures [23]. In the final step, given the optimal number of cluster (*k*) determined by dendrogram and NbClust, *k*-means clustering algorithm was conducted to the data set in order to identify the center of the cluster to show the standardized courses of the MC.

For the validation of the clusters, silhouette index was computed and the plot was made.

### Statistical analysis

For cluster analysis, all the codes were written in R programming language with various R packages such as clustertend [24], factoextra [25], NbClust [23], and cluster [26]. The codes were run in RStudio (v1.3.1073) based on R for window program (v4.0.2). To assess the equality of variances for the cluster groups, Levene’s test was applied. Since there are no true labels available for cluster validation in this study, ANOVA was selected to verify whether there are significant differences between the clustered groups. One-way ANOVA with Bonferroni post-hoc test was used to determine whether the homogeneity of the variables was satisfied, otherwise One-way ANOVA with Welch correction and Games-Howell post-hoc test was conducted with 5% significance level. The proportions of distributions were calculated according to MC course clusters, sex, left and right sides of the mandible, and age. The equality of proportion of MC courses was analyzed according to sex and sides using Z-test (*P*<0.05).

## Results

### Subjects

A total of 429 subjects were included in this study. 216 were male (50.3%), and 213 were female (49.7%). The mean age was 38.2 years, and the age ranged from 18 to 81 years. A total of 858 hemi-mandibles, 429 (50.0%) pairs of the right and left mandibles, were analyzed. The age distributions were as follows: 18–19 years, 63 (14.7%); 20–29 years, 103 (24.0%); 30–39 years, 74 (17.2%); 40–49 years, 64 (14.9%); and 50–59 years, 64 (14.9%); over 60 years, 61 (14.2%).

### Clustering of the 3D mandibular canal course

**Fig 3:**
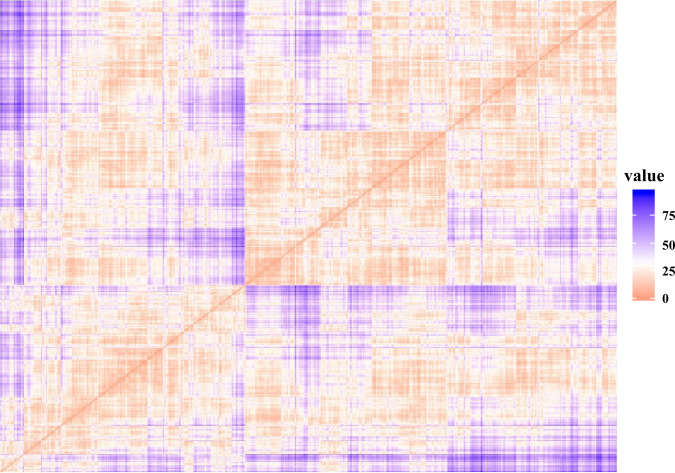
Dissimilarity matrix visualizing the clustering tendency of the parameters. Orange shows low dissimilarity level and purple shows high dissimilarity level.

The results of cluster tendency in normalized parameters were presented by Hopkin’s statistics value and dissimilarity matrix. Calculated Hopkin’s statistics for the MC parameters was 0.2096047, far below the H threshold of 0.50. Fig 3 displays the dissimilarity matrix that obtained from VAT algorithm. Orange shows low dissimilarity level and purple shows high dissimilarity level. In the diagonal direction, when the parameter is itself, i.e., the distance between observations is zero, it is displayed in red. Objects assigned to the same cluster in a dissimilarity matrix are displayed consecutively, clustering trends can be detected with rectangular-shaped blocks located along diagonal lines.

**Fig 4:**
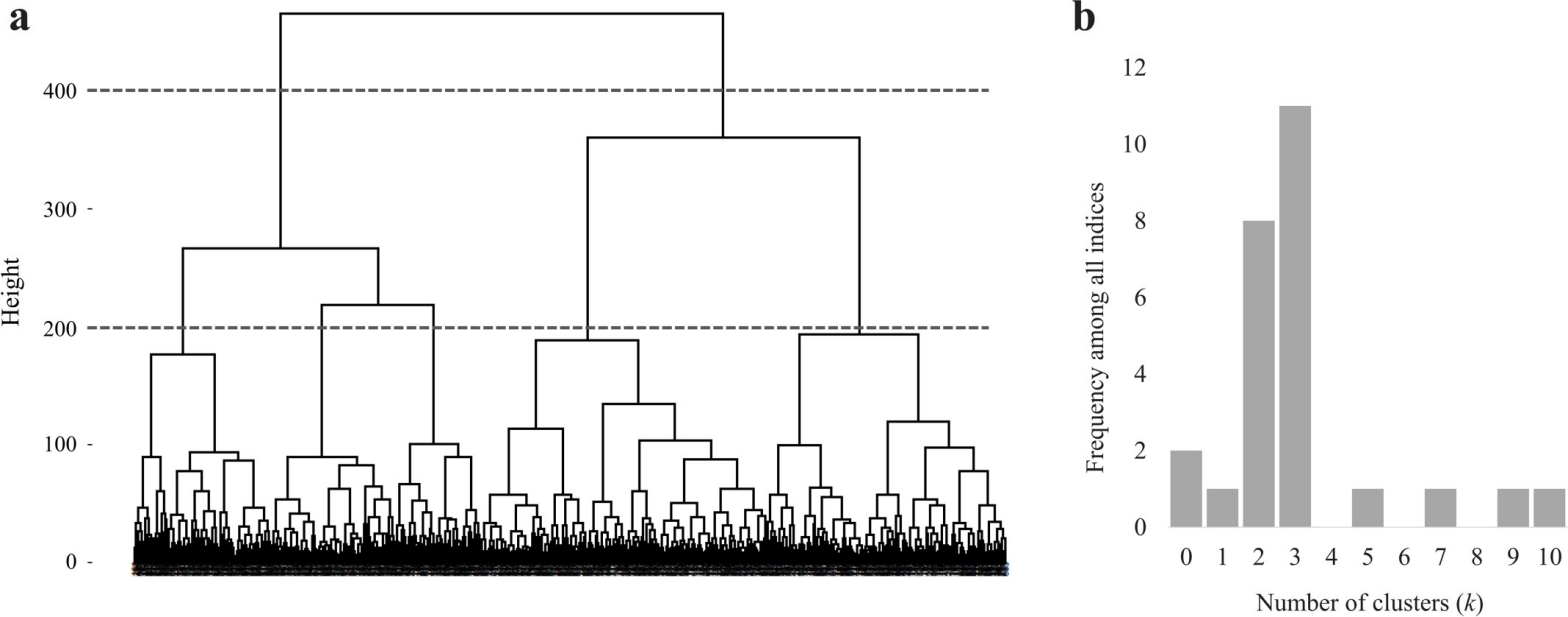
Determination of the optimal number of clusters using dendrogram (a) and NbClust (b). (a) The vertical axis represents the distance between clusters and the horizontal axis represents individual variables. The dotted lines show an example of determining the optimal number of clusters. The clusters are classified into 2 and 5 groups at height 400 and 200 respectively. (b) Frequency distribution of NbClust indices for the number of clusters.

The optimal number of clusters was determined as three by dendrogram and computing method. Fig 4A shows a dendrogram that was generated as the result of hierarchical clustering conducted for the classification of 3D MC course. Considering the variation of the MC course pattern, the range of the optimal number of clusters can be estimated from two to five clusters at 400 and 200 height level, respect. In Fig 4B, 11 indices suggest three as the optimal number of clusters.

Based on the result of *k*-means cluster analysis, the means and standard deviations of three clusters of the 3D MC courses were presented on Table 1. There were statistically significant differences among the means of the three clusters both in vertical and horizontal parameters. Cluster 1 showed the most rapid vertical change, ranging from 27.9 ± 4.5% in S1 to 74.0 ± 9.9% in S4. Conversely, cluster 2 showed the lowest vertical increase rate from S1 (23.5 ± 3.8%) to S4 (48.6 ± 7.7%). On the other hand, cluster 3 showed the largest distribution range in horizontal view, from 53.9 ± 8.1% to 65.9 ± 6.3%, whereas cluster 1 had only 4.6% variation range.

**Table 1:** Mean and standard deviation of 3D mandibular canal courses according to cluster type.

	Cluster 1	Cluster 2	Cluster 3	*P-value*
	(N = 223)	(N = 361)	(N = 274)	
Vertical parameters (%)				
S1	27.9 ± 4.5 ^a^	23.5 ± 3.8 ^b^	24.5 ± 3.9 ^c^	0.001***^☨^
S2	33.4 ± 5.0 ^a^	26.1 ± 4.4 ^b^	27.9 ± 4.2 ^c^	0.001***^☨^
S3	45.0 ± 6.1 ^a^	32.2 ± 5.4 ^b^	34.7 ± 4.9 ^c^	0.001***^§^
S4	74.0 ± 9.9 ^a^	48.6 ± 7.7 ^b^	54.3 ± 8.4 ^c^	0.001***^§^
Horizontal parameters (%)				
S1	65.2 ± 8.3 ^a^	70.0 ± 7.7 ^b^	62.3 ± 8.0 ^c^	0.001***^☨^
S2	69.2 ± 7.0 ^a^	74.4 ± 6.0 ^b^	65.9 ± 6.3 ^c^	0.001***^☨^
S3	69.8 ± 7.0 ^a^	73.5 ± 5.5 ^b^	62.9 ± 5.8 ^c^	0.001***^§^
S4	68.9 ± 10.3 ^a^	69.6 ± 7.6 ^a^	53.9 ± 8.1 ^b^	0.001***^§^

Values are presented as mean ± standard deviation in ratio.

^☨^ P-value by One-way ANOVA at α = 0.05.

^§^ P-value by One-way ANOVA with Welch correction at α = 0.05.

S1 to S4 represent four sites measured vertical and horizontal parameters.

^a-c^ Superscripts on the same row indicate significant statistical differences (Bonferroni post-hoc test for S1 and S2, and Games-Howell post-hoc test for S3 and S4)

*, *P* < .05;

**, *P* < .01;

***, *P* < .001.

**Fig 5:**
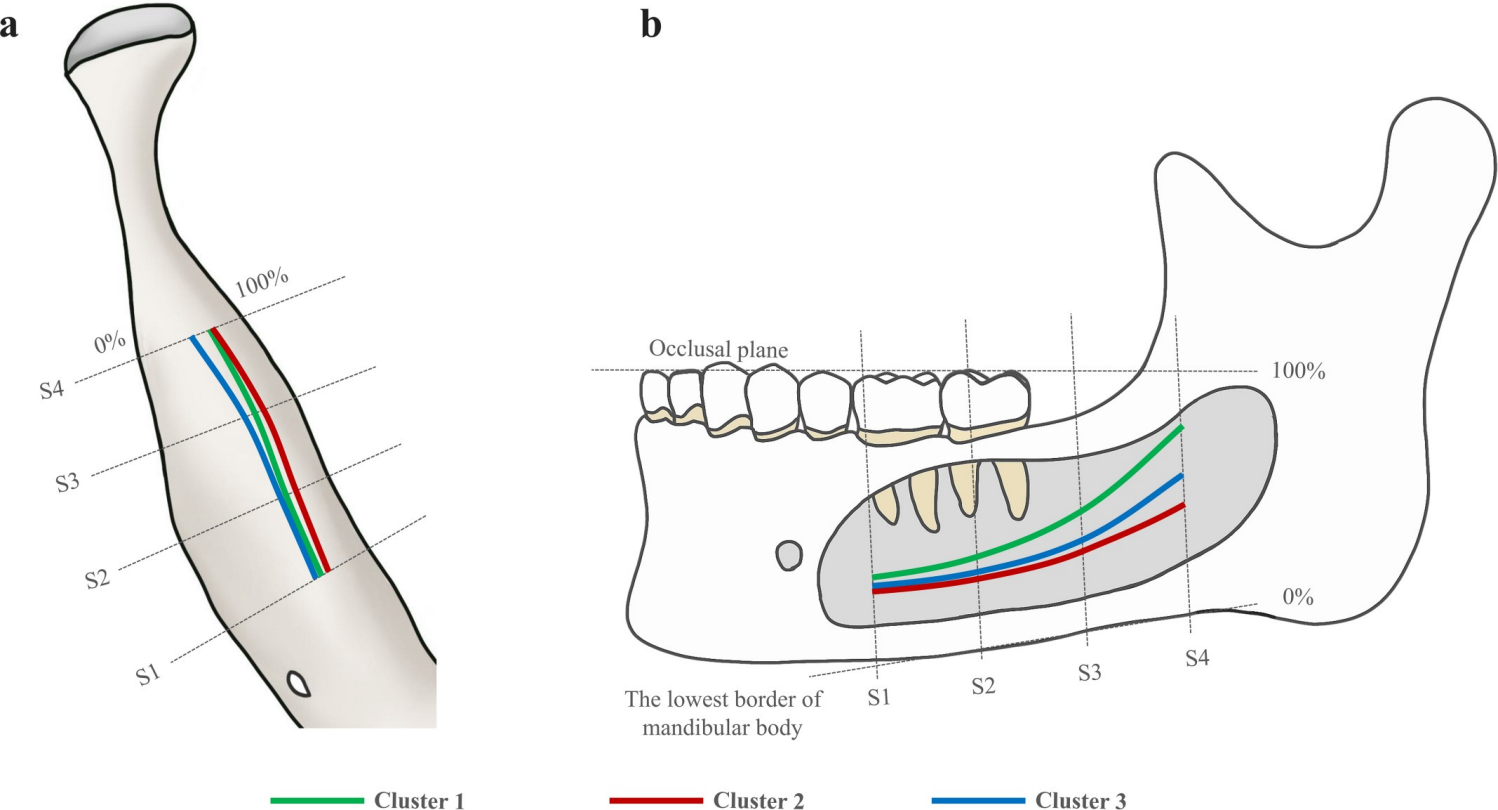
Illustrations of three types of the three-dimensional mandibular canal course in axial (a) and sagittal view (b). Cluster 1 shows traveling towards the lingual side with a steep vertical slope. Cluster 2 shows the closest to the lingual side and lowest vertical slope. Cluster 3 shows a buccal curvature with gradually increasing vertical slope in the posterior area.

Fig 5 shows the superimposed courses of all three clusters, making it easy to compare relative positions of the 3D MC courses. Cluster 1, green line, showed a smooth line running towards the lingual side in the axial view (Fig 5A) and a steep slope in the sagittal view (Fig 5B). Cluster 2, red line, ran in an almost straight line closest to the lingual and inferior border of mandible (Fig 5A and 5B); it showed the least change in the driving course compared to other MC course types. Cluster 3, blue line, showed the pathway with a bent buccally in the axial view and an increasing slope in the sagittal view in the posterior area (Fig 5A and 5B); this type showed a larger change in the posterior area.

### Distributions of the 3D mandibular canal courses

**Fig 6:**
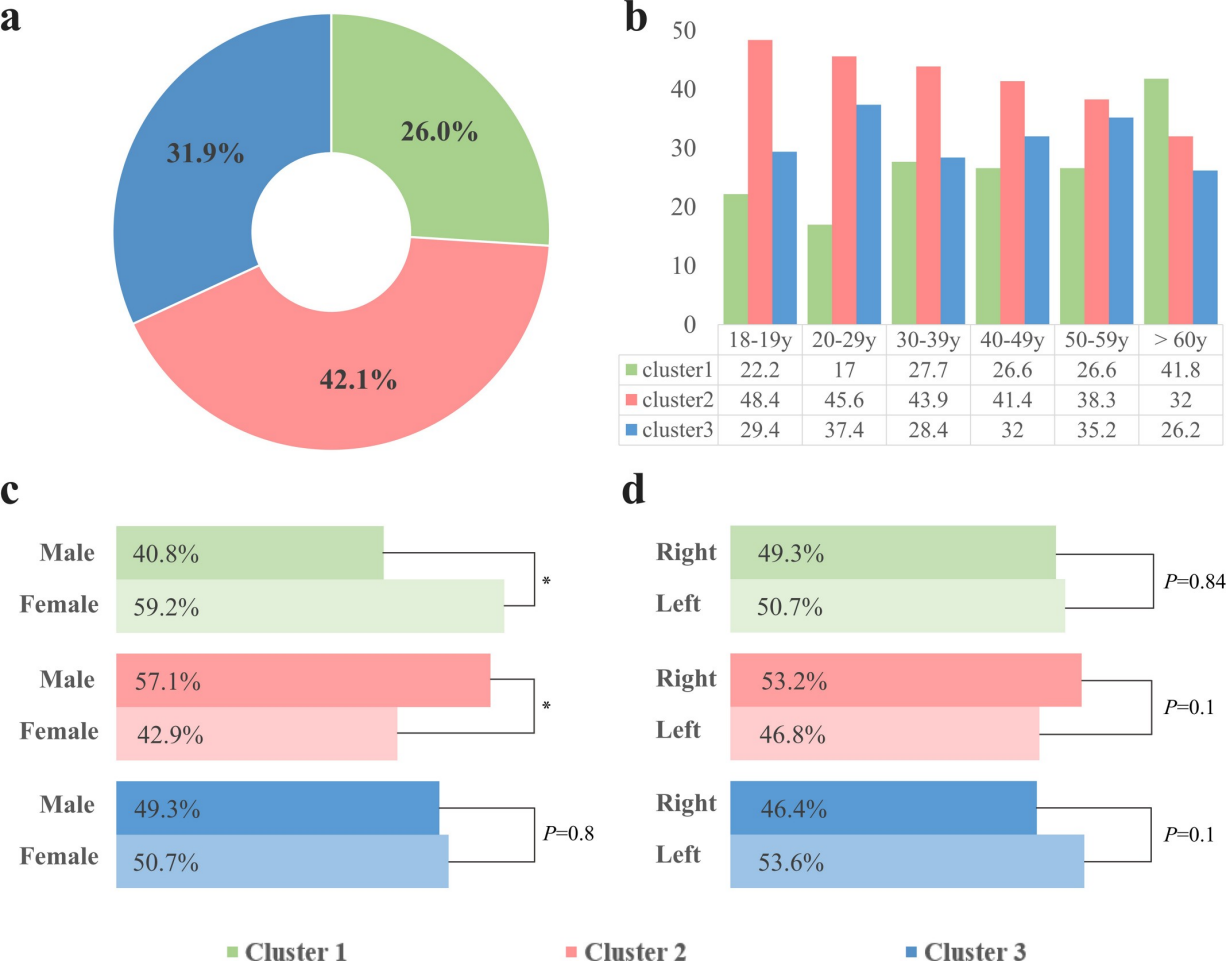
Distribution of the three-dimensional mandibular canal course by cluster. (a) Distributions of three clusters, (b) Distribution of three clusters for each age group, (c) Sex distribution of each cluster (*P*-value by Z-test at α = 0.05. *, *P* < .05.), and (d) Right and left distribution of each cluster.

Distributions of the mandibular canal courses according to clusters, age groups, sex, and right and left sides are displayed in Fig 6. Fig 6A and 6C shows the distributions of classified clusters and sex within clusters. Cluster 1 had the least distribution (26.0%). In this cluster, females accounted for 59.2% and males, 40.8%. On the other hand, cluster 2 showed the widest distribution of driving courses (42.1%), and males were more widely distributed (57.1%) than the female group (42.9%). Cluster 3 comprised similar ratio of male and female cases and accounted for 31.9% of the total distribution. For all three clusters, distributions of the right and left sides did not show a statistically significant difference (Fig 6D). Distribution of cluster was inversely proportional to age of subject, and cluster 1 showed the highest distribution (41.8%) in the age of 60 or older (Fig 6B).

## Discussion

Standardized information of anatomical structure is one tool that facilitates communication among clinicians [27]. Existing classifications were commonly developed through visual assessment of cadaveric dissections or radiographs, and has been used in practice to date [28]. However, the inevitable variability in interpretation among experts proves that the use of previous classifications as standard information may be limited. In medical field, cluster analysis has been widely utilized to objectively distinguish the given data such as gene and patient information, but less applications are conducted for the anatomical structure classification [29, 30]. In this study, cluster analysis was applied to classify 3D MC courses and to present standard types of MC courses.

Based on the results of current study, three representative 3D MC course types were derived from the cluster method, and the presented courses can be compared with the outcomes of existing studies on the MC course classification. For examples, Worthington classified the vertical MC courses into three categories: an anteriorly gentle and posteriorly progressive rising curve, a steep ascent, and a catenary-like canal [8]. Many researchers have used this classification or modified it to present the MC course and its distribution [13, 31–33]. In comparison, the sagittal view of the 3D MC courses visualized in this study, a steep vertical slope (cluster 1) and the straight path closest to the inferior border (cluster 2) were presented similarly with the Worthington classification, while a catenary-like pathway was not found. This phenomenon is the same for the axial view of the MC course. The individual MC courses clustered in the current study differed from the known horizontal MC courses [33–35]. Discrepancy between the results of previous studies and this study may arise from different methodologies. Most of the research were confined to 2D analysis of the MC course, viewed from either axial or sagittal view. In other words, the classification result was presented after considering the vertical and horizontal location of MC, respectively. On the other hand, in the present study, both horizontal and vertical parameters of the MC course were considered together by cluster algorithm firstly; and then for each cluster, 2D axial and sagittal images of the MC courses were obtained. To accurately classify the anatomical structure, an analysis taking into account the 3D information is required.

Interestingly, if the data of this study were intently divided into a larger number of clusters, the MC course would be grouped into more diverse clusters, and the MC courses reported in previous studies (e.g., catenary-like courses) may also be found among them. However, in the current study, the optimal number of clusters was established as three using dendrogram and computing method to derive objective classification. Although statistical representativeness is reduced, if clinically necessary, observers can classify MC courses in detail with the k-means cluster algorithm.

Considering the distribution trend of the MC courses according to age group, cluster 1 showed the highest value in the group over 60 years old, and cluster 2 gradually decreased as age increased. This may be due to age-related factors such as tooth loss and attrition that cause a decrease in MnOP levels [36, 37]. Levine et al. [38] reported that age and race were related to the MC location, so further study of the MC courses by race using cluster analysis is needed. As a result of analyzing the left and right distribution, no significant difference was found in accordance with the previous study, which reported that right and left side were not factors affecting the location of the MC [39].

Expert reliability is one of the major issues to be addressed in anatomy structure classification task [40, 41]. Although ensuring expert qualifications through training is widely carried out to improve reliability, even so, subjective evaluation among observers is not completely overcome [42]. Given the high variability in the anatomical structure, expert disagreement on cases where grouping is ambiguous can affect the classification results. In this study, the cluster algorithm was designed to divide groups according to the similarities between all given variables, so it automatically classified debatable MC course cases without observer variability and present representative results. This unsupervised machine learning technique can be applied to analysis of various anatomical information that has been mainly performed by experts, and the presented standard information can be used in divergent fields such as dental treatment planning, forensic usage, and model generation for reconstructive surgery.

The cluster analysis method that provides information on hidden structures by analyzing given variables and gathering them into homogeneous groups with similar characteristics, have actively applied in the healthcare field. Most of the previous studies have obtained information about the characteristics of clustered patients by analyzing various factors such as symptom, behavior, disease and knowledge [30, 43–46]. Our study followed the general process of clustering analysis performed in previous researches, and there may be few novelties identifiable during the analysis method. However, the contribution of our study can be found in the application of clustering techniques for the objectively classification of the anatomical structures, one of the major clinical problems to be solved. Since the cluster analysis algorithm is designed to present the optimal cluster based on the similarity between variables without bias against the measured variables, objective and reproducible classification is possible even in ambiguous cases.

The limitation of this study was that it did not include areas near the mental and mandibular foramen. Regarding that the anterior loop region is accompanied by high anatomical variation [47], it is challenging to measure the area in a standardized way in all patients. However, even if the number of additional parameters was updated as new areas were included, cluster analysis would make it easy to re-classify MC course types, considering the similarity of all given variables. The difficulty of conducting external cluster validation for this task may be another limitation. However, since there is no consensus on classification labels of the MC course, we demonstrated statistical significance through ANOVA analysis on clustered groups [48].

In conclusion, the 3D MC courses were automatically classified as three types through cluster analysis and each classified course was statistically distinct from other clusters. This study demonstrates the feasibility of using cluster analysis for measured parameters in medical imaging. Cluster analysis enables the unbiased classification of the anatomical structures by reducing observer variability and can present representative standard information for each classified group. Looking forward, further attempts with various anatomical structures that did not reach consensus due to the variability of the examiners could prove the benefits of this method.
